# Supplementary material for: Acceptance and compliance with micronutrient powder (MNP) among children aged 6–23 months in northern Nigeria
Source: PLOS Glob Public Health. 2022 Oct 17;2(10):e0000961. doi: 10.1371/journal.pgph.0000961 (PMC10022258; doi:10.1371/journal.pgph.0000961)
Supplement: S1 Dataset — (ZIP) [file pgph.0000961.s008.zip › S8_Dataset/In-depth Interviews.zip!/In-depth Interviews/11072015_A_GA_AD_2_CG.pdf]

INTERVIEW WITH CAREGIVER AT DADIRI COMMUNITY IN GANYE LOCAL GOVERNMENT AREA, ADAMAWA STATE

I: Describe for me the people in your immediate family living in your household

R: There are four of us in this household. This is a large household I mean it is an extended family house. There is my husband, our 2children and I in my immediate family.

I: Tell me more about your children's gender and age

R: My older child is 4years and the younger child is 17months old. When I started giving her the MNP she started purging, I told my husband's elder brother about it and he encouraged me to continue. I continued for a few more days but she was still purging so I took her to the hospital. I didn't tell them I was giving her MNP and it might be the one that caused the purging. They gave us medicine for diarrhoea and vomiting; now she is feeling better. Let me show you the syrups they gave us. Yesterday she had diarrhoea again and I gave her the medicine but now I understand that it is not necessarily the MNP that made her to purge.

I: We have heard of food challenges for young children in this setting related to the season. Could you talk about these challenges?

R: What I understand is that it may have been the food that the child ate that made her to vomit and purge. The other day they cooked cassava flour meal with fish that I cooked the previous night; I wasn't at home because I had to rush to the hospital to see my mother who was admitted. They also got some soup from their grandmother next door (moringa soup without groundnut paste in it), I think that is what made her to vomit and purge. I gave her the MNP again but she had diarrhoea again so I stopped. I just don't understand what is wrong.

I: Could you talk about the food challenges for young children in this community

R: Ideally the way I take care of my children, for example my older child was 2years old before I stopped breastfeeding her and she was eating a variety of food and also drinking kunu before I weaned her. My younger child is also eating an drinking while still

breastfeeding but I think she has stomach problem because there are times when she eats and suddenly has diarrhoea or vomits

I: Could you discuss how food challenges affect infant and young feeding in this community

R: before and after weaning a child, I always make kunun gyada for them with guinea corn. I also give them eggs, bananas and so on.

I: could you talk about the challenges related to season

R: We face most of these challenges during the rainy season because we might not get nutritious food to give the children. Sometimes even if it is available we do not have the money to buy the nutritious food.

I: Tell me about the coping mechanism to these challenges

R: As for my children, they like eating boubab soup, okro soup and sesame leave soup. They don't like foods such as rice and stew because even if I give them they don't eat it. Yesterday when I cooked ayoyo soup, my younger daughter ate it so much

I: One thing we heard in other interviews is that caregivers often give their children 6-23months "tea". Could you define/explain "tea"?

R: I do give her tea but she rarely drinks it. It seems she doesn't like it.

I: Could you define tea

R: I make the tea with bournvita and milk. I boil water without adding lipton, then I put it in flask. Whenever I want to make the tea, I put the beverages in a cup then add the hot water. I usually serve it with bread but she doesn't like it. It is my older daughter that eats it because after I weaned her she had refused kunu, so we usually give her tea with bread each morning

I: Now I want us to talk about the MNP that you received. Could you tell me about your experience with the product?

R: Well if my child had not started vomiting breast milk and purging, I would have seen more impact on the child but as the case is now I had stopped giving her the MNP since she

started purging and vomiting breast milk. To the best of my knowledge this MNP is really important in a child's health. My assumption now is that the MNP met some disease in her stomach which it is trying to repel that is why she is purging. From the little she has taken, she has added weight; she has increased appetite and falls ill less frequently unlike before.

I: Tell me more

R: They told us to administer the MNP with semi solid food like kunu and I had made kunu that morning and put in flask. The MNP is not bitter; I had tasted it before giving her the first day. I gave her the MNP and after a little while I breastfed her that is when she started vomiting and this has never happened to her. Then in the night, she started running a fever so I asked my husband if it could be the MNP but he said he didn't know. I carried her from the bed and she was standing by the bedside when she suddenly started purging (watery stool). The next day I just took her to the hospital where she was given some injections and drugs. She is feeling better now

I: uh-huh

R: I have not paid much attention to the pattern of eating though but sometimes immediately you feed a child, he might see something and want to eat or would like to breastfeed immediately after a meal. These might be what caused the vomiting. When they brought the MNP, they told us to give our children first thing in the morning before any meal in kunu or soup.

I: Some caregivers mentioned that it was hard to store the MNP without it being taken by others or eaten by rodents. Did you have any similar experience?

R: I keep the MNP in her basket. As you can see I have only given her 5 out of the packet, I had safely kept it so there has not been taken or eaten by rodents. Even inside the basket, I kept it behind a pack of cassettes.

I: Some mothers indicated that it might be hard to remember to use the MNP. Did you have a similar experience and what did you do to remember?

R: I gave my child the MNP in kunu except for one time when I gave her in tea. I have never forgotten to give it in the few days I did but when he started diarrhoea and vomiting I stopped it

I: could you tell me any specific solution to address the issue of remembering to use the MNP?

R: Well my advice to mothers is there is no product that is brought to us for immunization that has not benefitted our children. I will advice mothers to put in enough effort to ensure their child gets the best. If these kinds of product are brought we should accept it wholeheartedly because it is not here to harm us but rather to benefit us. Moreover if it were something harmful, then we would have seen its negative effect once we administer it to our children.

I: Let's talk now about the child's feeding and response to MNP

R: She didn't refuse the MNP from the beginning and she takes it in kunu and drinks the whole kunu when I mix it with the MNP. I gave her first thing in the morning

I: Could you tell me to what extent the child likes or dislikes the taste of MNP

R: She didn't complain of the taste because it doesn't have any specific taste; I had tasted it the first day I had given her the MNP. I serve a little portion of kunu then add the MNP and give her.

I: You mentioned some benefits of MNP earlier; could you talk more about on how MNP impacted on your child's diet?

R: She consumes more varieties of foods. She has increased appetite and consumes more food. She consumes more breast milk too. She has not had fever or headache since I gave her the MNP; she used to have fever frequently before I gave her the MNP.

I: In other communities we noticed that sharing of the MNP occurred. Could you talk about any sharing of the MNP when you used it?

R: There was no sharing because my older daughter knows I don't joke when it comes to medicine so she has never asked for it. She doesn't touch it when I keep it. I was the only

one who tasted it and it was the first day and I just used my finger to taste it to be sure it is not bitter.

I: Could you tell me about the leftovers of the MNP being consumed by others

R: She has never left the kunu mixed with MNP, she always finishes it

I: Could you tell me whether 60sachets will be enough for 60days?

R: I believe the 60sachets will be enough for the 60days and some people might forget to give a child or might not use it appropriately. Everyone has their way of thinking and a different opinion. Had it not been for the diarrhoea I would have given my child the whole 60sachet because I adhered to the instruction given to us on how to use it appropriately.

I: now that you have used the MNP, could you talk about how you perceive it?

R: I believe it is medicine for immunization for children. When given to them, they will eat well and they will more strength. They will also be protected from illnesses for children. See as my daughter does things with full energy since I gave her this MNP

I: Could you tell me whether you would purchase this MNP if it were cheaply on sale in this community

R: Some people will not even go near it if it is for sale but if it is free everyone will come out and collect it. As for me I have seen its importance and I can buy it

I: Could you tell me how much you will be willing to pay for each sachet

R: I don’t know

I: Please tell me your opinion on how much you can pay for each sachet. Your suggestions are important to us

R: Health is the most important thing in this world. In my opinion if this is to be sold to give their child for improved health, better diet and protection against illness, then the packet can be sold for 200naira each and each sachet should be sold at 10naira. This is what I am willing to pay.

I: You are giving me great information. Now tell me some ways that you believe would be effective for promoting MNP to caregivers in this community so that they would comply with using it appropriately?

R: Is it only in this community or everywhere that you will distribute the MNP?

I: I want to know the effective way of promoting MNP in this community

R: I don't know about others but I believe this is a form of immunization and if the community members are enlightened about it as immunization against illnesses and a child will have a better appetite and be healthy when given the MNP. If they are well enlightened then they will comply with its use appropriately.

I: Could you tell me what people influence your opinion and knowledge about feeding practices

R: My husband's older brother is the community leader here in Dadiri, he has been the one who encourages us to use such products. Whenever something is brought he gathers the community members informing them about it. Even the day that this MNP was brought, he came to call me by himself to take my child to the people that came to distribute the MNP

I: Could you tell me who influences your decisions regarding feeding your baby the MNP?

R: When I collected the MNP my husband was in the farm so when he got back I showed him and told him about it, he asked what it is meant for and I told him it is going to help our child and we were told to give them in semi solid food like kunu. He didn't say anything else so I continued to give the child and he didn't say anything about it too. He didn't say to give her or not to, he is just going about his activities. I am the one who takes care of the children, he just ensures he provides what we need even if any of the children are ill, he will only give money to go to the hospital.

I: Could you tell me where would be a good place to disseminate health information related to MNP?

R: Usually there is a place some nurses' stay in front of the late community leader before the leaders was given to my husband's older brother who is also our neighbour. There is a

big mango tree in front of his house where they gather the community members; I mean in front of the late community leaders house. Whenever the people are called upon, they will gather there. The community members are always ready to accept things that will help them and their children; the day the MNP was distributed here, some people were accusing the community leader of sidelining them but he explained to them that it is only meant for children 6-23months and it is a trial phase. After the trial there will be massive distribution.

I: Could you tell me who, in this community, you trust the most for receiving messages about infant and young child feeding?

R: There used to be a clinic here but it's not functional now. The nurses go house-to-house round the village and even the community leader's son is a healthcare worker

I: tell me more

R: They are still working on the clinic that is why the nurses stay in front of the late community leader's house. The healthcare worker, the community leader's son, can enlighten us on infant and young child feeding and we will listen to him because we trust him and he will never encourage us to engage in anything harmful

I: Could you tell me what form of media you prefer for receiving MNP message?

R: It is not everyone that listens to the radio, some do but some don't. Most of the women in this community go to the farm; it's only a few that stay at home. My advice is to send a message whether written or verbal; those who can't read can be told about it too. The most appropriate is to go through the community leader because some of these our people will say what you know about taking care of a child but some will listen and readily accept it.

I: Could you tell me the preferred ways of receiving education regarding IYCF and MNP?

R: Well there have been immunization programs in this community and we always gather to receive the medicine, they were here not long ago to give our children vitamin A, medicine for worms and polio vaccination.

I: Please tell me specific examples of any successful health education campaign in this community. What made it successful?

R: Whenever such is brought they go to the community leader informing him about whatever it is, he then send for the community members especially those who have the targeted age children. The community members gather in front of the community leader's house to be enlightened and receive the immunization. It has been successful this way

I: Please tell me the best community channels to distribute this product

R: The people that came to distribute this MNP came directly to the community leader and he welcomed them and suggested the ones to be given the MNP. We were called and enlighten about it and they gave us the MNP. They emphasized on giving the MNP in a semisolid food and ensure it was given each morning.

I: Ok. Could you tell me the best channel to distribute when the MNP is brought in the future?

R: The community leader should be put in charge of the distribution because the nurses don't even live around here; they come from the general hospital at Ganye II and they leave around 1 or 2pm.

I: I want to hear some specific messages you think would effectively promote the product

R: Some caregivers have bad opinion on such things but some will come and collect when they get proper enlightenment. Some will collect and just keep it without using it and will tell you they are using it. Some will argue that it has no benefit while others will not use it until someone else does and they see the benefit of the product. The general fear around here is that this kind of product cause infertility to children or it is bad in some way

I: You mentioned that some caregivers might be reluctant to accept this product, what message should be used to explain MNP to the caregivers?

R: I would tell them that when they give their child this MNP, it would increase their appetite because I have seen how my child eats now. She eats more varieties of food now unlike before, she has more energy and plays a lot. This product is meant to improve our children's health and I urge my fellow mothers to use this product for the betterment of our children because it will protect them from so many illnesses.

If they are told this, they will accept the product and use it for the 60days that is prescribed

I: Please tell me what specific message will be effective to promote its use but limit the sharing of the MNP

R: As I have said earlier, when the community members are gathered in front of the community leader's house, it will be distributed there

I: Some people will use it but how do we limit the sharing?

R: Enlightenment is the key. Since the MNP is meant for children under 2years, they should be told that it will not be of any benefit to a child more than 2years old.

I: Finally, is there anything else you can share about your experience with this product?

R: I gave my child the MNP for 5days only, even though she had diarrhoea and was vomiting, I had seen some improvement in my child. Before giving her the MNP she only breastfeeds and doesn't eat other foods but now she eats so many varieties of food and is full of energy. She suffered fro constant fever but now she is ok after giving her the MNP

I: Could you recommend anything else to help develop an effective program?

R: My recommendation is to enlighten the whole community through the community leader and his assistants. The community members need to be encouraged to come out and collect this product for their children

I: Tell me more

R: I have a question, is diarrhoea and vomiting of breast milk part of the side effect of the MNP? I have concluded that it might have been the food that she consumed that caused the diarrhoea.

I: Is there anything else you would like to add?

R: No.

I: Thank you for your time and the information
